# Influence of starch deficiency on photosynthetic and post-photosynthetic carbon isotope fractionations

**DOI:** 10.1093/jxb/erz045

**Published:** 2019-02-09

**Authors:** Marco M Lehmann, Shiva Ghiasi, Gavin M George, Marc-André Cormier, Arthur Gessler, Matthias Saurer, Roland A Werner

**Affiliations:** 1 Forest Dynamics, Swiss Federal Institute for Forest, Snow and Landscape Research WSL, Zuercherstrasse, Birmensdorf, Switzerland; 2 Institute of Agricultural Sciences, ETH Zurich, Universitaetstrasse, Zurich, Switzerland; 3 Institute of Molecular Plant Biology, ETH Zurich, Universitaetstrasse, Zurich, Switzerland; 4 GFZ – German Research Centre for Geosciences, Geomorphology, Organic Surface Geochemistry Lab, Telegrafenberg, Wissenschaftspark Albert Einstein, Potsdam, Germany; 5 University of Oxford, Department of Earth Sciences, Ocean Biogeochemistry Group, South Parks Road, Oxford, UK; 6 Institute of Terrestrial Ecosystems, ETH Zurich, Universitaetstrasse, Zurich, Switzerland

**Keywords:** Assimilates, carbon allocation, carbon storage, ecophysiology, isotope effect, metabolic fluxes, non-structural carbohydrates (NSCs), plant respiration, starch-deficient mutant (SDM), starchless mutant

## Abstract

Carbon isotope (^13^C) fractionations occurring during and after photosynthetic CO_2_ fixation shape the carbon isotope composition (δ^13^C) of plant material and respired CO_2_. However, responses of ^13^C fractionations to diel variation in starch metabolism in the leaf are not fully understood. Here we measured δ^13^C of organic matter (δ^13^C_OM_), concentrations and δ^13^C of potential respiratory substrates, δ^13^C of dark-respired CO_2_ (δ^13^C_R_), and gas exchange in leaves of starch-deficient plastidial phosphoglucomutase (*pgm*) mutants and wild-type plants of four species (*Arabidopsis thaliana*, *Mesembryanthemum crystallinum*, *Nicotiana sylvestris*, and *Pisum sativum*). The strongest δ^13^C response to the *pgm*-induced starch deficiency was observed in *N. sylvestris*, with more negative δ^13^C_OM_, δ^13^C_R_, and δ^13^C values for assimilates (i.e. sugars and starch) and organic acids (i.e. malate and citrate) in *pgm* mutants than in wild-type plants during a diel cycle. The genotype differences in δ^13^C values could be largely explained by differences in leaf gas exchange. In contrast, the PGM-knockout effect on post-photosynthetic ^13^C fractionations via the plastidic fructose-1,6-bisphosphate aldolase reaction or during respiration was small. Taken together, our results show that the δ^13^C variations in starch-deficient mutants are primarily explained by photosynthetic ^13^C fractionations and that the combination of knockout mutants and isotope analyses allows additional insights into plant metabolism.

## Teaser

Carbon isotope composition of starch-deficient mutants is primarily explained by photosynthetic ^13^C fractionations, and thus plant physiological responses to changes in carbon storage, rather than by post-photosynthetic ^13^C fractionations.

## Introduction

Short-term variation in the carbon isotope composition (δ^13^C) in plant-respired CO_2_ and in the related respiratory substrates is of wide interest for plant ecophysiological studies investigating carbon allocation (Brueggemann *et al.*, 2011; Hagedorn *et al.*, 2016; Galiano Pérez *et al*., 2017), for reconstructions of plant functional responses to climatic conditions (Ehleringer *et al.*, 1997; Gessler *et al.*, 2014; Ehlers *et al.*, 2015), and for the plant biochemical community measuring and modeling metabolic carbon fluxes (Werner *et al.*, 2011; Szecowka *et al.*, 2013; Sweetlove *et al.*, 2014). The enzymatic and diffusional carbon isotope fractionations during photosynthesis are well known and can be mathematically modeled (Farquhar *et al.*, 1989), with the ratio of leaf internal (*c*_i_ or, more precisely, chloroplastic *c*_c_) to atmospheric (*c*_a_) CO_2_ concentrations as a key parameter for the ^13^C depletion of organic matter relative to atmospheric CO_2_. The *c*_i_/*c*_a_ ratio is regulated by plant physiological parameters such as the photosynthetic assimilation rate (*A*_n_) and stomatal conductance (*g*_s_) in response to environmental conditions (Brugnoli *et al.*, 1988; Scheidegger *et al.*, 2000). Subsequently, the photosynthetic assimilates, such as leaf (transitory) starch and sugars, and downstream metabolites undergo additional, so-called post-photosynthetic (synonym: post-carboxylation) ^13^C fractionation processes during the maintenance of plant metabolism, respiratory processes, carbon allocation, and/or biosynthesis of various primary or secondary metabolites (Werner and Gessler, 2011; Werner *et al.*, 2011). However, given that post-photosynthetic ^13^C fractionation processes can overlap with each other or with the *c*_i_/*c*_a_-driven photosynthetic ^13^C fractionations (Brandes *et al.*, 2006), their magnitude and influence on short-term δ^13^C variation in leaf dark-respired CO_2_ (δ^13^C_R_) and respiratory substrates is still under debate (Werner and Gessler, 2011; Gessler *et al.*, 2014).

**Fig. 1.**
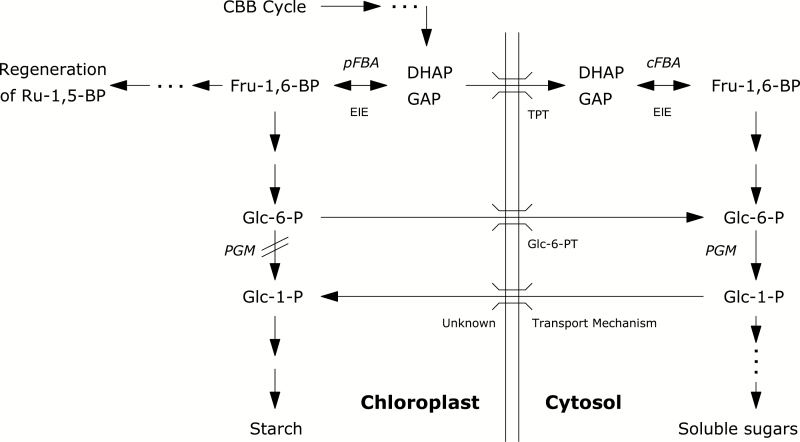
Reaction scheme for potential post-photosynthetic ^13^C fractionations via pFBA in *pgm* mutant plants. Photosynthetic triose phosphates from the CBB cycle are linked via the pFBA reaction to starch biosynthesis in the chloroplast of wild-type plants. An equilibrium isotope effect (EIE) on the pFBA causes the triose phosphates used for starch biosynthesis to be more ^13^C enriched than those exported to the cytosol for sugar (e.g. sucrose) biosynthesis (Gleixner and Schmidt, 1997). In contrast, the main route for starch biosynthesis is blocked in *pgm* mutants. The surplus of ^13^C enriched triose phosphates in the chloroplast might be transported to the cytosol, potentially causing a ^13^C enrichment in sugars. Additional bypass reactions for the starch residue in *pgm* mutants are indicated. CBB cycle, Calvin–Benson–Bassham cycle; pFBA and cFBA, plastidic and cytosolic fructose-1,6-bisphosphate aldolase; PGM, phosphoglucomutase; TPT, triosephosphate translocator; Glc-6-PT, Glc-6-phosphate translocator; DHAP, dihydroxyacetone phosphate; GAP, glyceraldehyde 3-phosphate.

One of the most important post-photosynthetic ^13^C fractionations is probably related to an equilibrium isotope effect on the plastidic fructose-1,6-bisphosphate aldolase (pFBA) reaction (Brugnoli *et al.*, 1988; Gleixner *et al.*, 1993; Gleixner and Schmidt, 1997). This isotope effect potentially explains the heterogeneous ^13^C distribution in carbohydrates that has been observed in glucose derived from C_3_ beet sucrose and C_4_ corn starch (Rossmann *et al.*, 1991) or from transitory starch in potato and beet leaves (Gleixner *et al.*, 1993; Gilbert *et al.*, 2012). The pFBA reaction reflects a metabolic branching point favoring ^13^C in fructose-1,6-bisphosphate under equilibrium conditions (Fig. 1). This ^13^C enrichment is passed to transitory starch (or ribulose-1,5-bisphosphate), whereas the ^13^C depleted triose phosphates are exported into the cytosol for sugar biosynthesis (e.g. sucrose) and glycolysis. Such a mechanism probably explains why transitory starch has been found to be more ^13^C enriched than sugars in beet leaves (Gleixner *et al.*, 1993). The pFBA reaction has been suggested to cause diel δ^13^C variation in sugars and starch (Brugnoli *et al.*, 1988; Gleixner *et al.*, 1993; Goettlicher *et al*., 2006; Gessler *et al.*, 2007; Lehmann *et al.*, 2015), thereby also influencing δ^13^C_R_ and the apparent respiratory ^13^C fractionation (*e*; Ghashghaie *et al.*, 2001, 2003; Werner *et al.*, 2009). In fact, diel δ^13^C variations in starch and in the organic acid malate have been identified as main drivers of diel changes in δ^13^C_R_ across different soil moisture and temperature conditions in potato plants (Lehmann *et al.*, 2015). Diel δ^13^C variation in leaf starch and sugars related to the pFBA reaction has also been found to partially influence the δ^13^C of phloem-exported sugars and source to sink ^13^C fractionations (Goettlicher *et al*., 2006; Gessler *et al.*, 2008; Barthel *et al.*, 2011), and thus ultimately the δ^13^C of tree-rings (Jäggi *et al*., 2002; Gessler *et al.*, 2014; Rinne *et al.*, 2015).

Both photosynthetic and post-photosynthetic ^13^C fractionations (e.g. via pFBA or respiration) are likely to be closely connected to leaf starch metabolism. Biosynthesis and storage of leaf starch occur in the chloroplast and are under strong control by the circadian clock, leading to significant diel variation in starch concentrations (Zeeman *et al.*, 2007; Stitt and Zeeman, 2012; Kolling *et al*., 2015). Starch functions as a highly flexible buffer molecule, balancing carbon supply and demand, and was identified as an important factor maintaining plant performance and growth (Huber and Hanson, 1992; Sulpice *et al.*, 2009). The leaf starch content also shows strong seasonal (Jäggi *et al.*, 2002) and species-specific variation (Ivanova *et al.*, 2008), and is additionally influenced by abiotic stresses such as drought (Lehmann *et al.*, 2015; Galiano Pérez *et al*., 2017; Thalmann and Santelia, 2017). The regulation and pathways of starch biosynthesis are still under debate (Streb *et al.*, 2009; Geigenberger, 2011).

To study starch biosynthesis and degradation, knockout mutants have proven to be invaluable (Stitt and Zeeman, 2012). However, mutant plants have only rarely been combined with stable isotope analysis. Pulses of ^13^CO_2_ have been applied to mutant plants to study biosynthetic pathways (Figueroa *et al.*, 2016; Baslam *et al.*, 2017), but very few studies have included carbon isotope analysis at natural isotope abundances to investigate δ^13^C differences among starch fractions or explore respiratory ^13^C fractionations (Scott *et al.*, 1999; Duranceau *et al.*, 2001). Thus, it is not yet clear whether plant mutants are helpful for elucidating ^13^C fractionations and the resulting ^13^C isotope signature of organic compounds.

Mutant plants lacking the expression of a functional plastidial phosphoglucomutase (PGM) have negligible starch concentrations. The enzyme catalyzes the reversible interconversion of glucose-6-phosphate to glucose-1-phosphate (EC 2.7.5.1) and is the main route responsible for starch production (Periappuram *et al.*, 2000; Streb *et al.*, 2009). The remaining starch residue in the *pgm* mutant has been suggested to be produced via cytosolic bypass reactions (Geigenberger, 2011). A number of *pgm* mutants have been isolated from species such as *Arabidopsis thaliana* (Caspar *et al.*, 1985), *Nicotiana sylvestris* (Hanson and McHale, 1988), *Pisum sativum* (Harrison *et al.*, 2000), and *Mesembryanthemum crystallinum* (Cushman *et al.*, 2008). The mutants feature a phenotype with a reduced growth rate (Caspar *et al.*, 1985; Huber and Hanson, 1992; Geiger *et al.*, 1995) but increased sugar concentrations during the day, higher leaf to phloem export, and enhanced root respiration (Brauner *et al.*, 2014).

Findings from some studies have indicated a reduction of assimilation rates in *pgm* mutants compared with wild-type plants (Caspar *et al.*, 1985; Huber and Hanson, 1992; Geiger *et al.*, 1995; Sun *et al.*, 1999). This may cause differences in photosynthetic ^13^C fractionations and thus changes in δ^13^C values of plant material, but the magnitude of such an effect has yet to be determined. On the other hand, the starch deficiency in *pgm* mutants probably influences post-photosynthetic ^13^C fractionations via pFBA. The reduced need for hexose-phosphates in the chloroplasts of *pgm* mutants causes an increased flux, in the form of triose-phosphates, to the cytosol, leading to soluble sugar biosynthesis. We therefore expect that the equilibrium isotope effect on the pFBA reaction is expressed to a lesser extent or not at all in the direction of starch, causing a ^13^C enrichment of cytosolic sugars and all downstream metabolites in *pgm* mutants compared with wild-type plants. Moreover, the PGM-knockout might also affect apparent respiratory ^13^C fractionations, given that the absence of starch metabolism may lead to changes in the δ^13^C of potential respiratory substrates (i.e. sugar, starch, and organic acids) and their pool sizes, thereby causing variation in δ^13^C_R_. The study of *pgm* mutants may therefore help improve our mechanistic understanding of ^13^C fractionations in plants.

Here we performed several experiments with *pgm* mutants and wild-type plants of different species. We hypothesized that the starch deficiency induced by the PGM-knockout leads to changes in (i) photosynthetic (via leaf gas exchange) and (ii) post-photosynthetic ^13^C fractionations (via pFBA and respiration) and thus to δ^13^C variation in organic matter, dark-respired CO_2_, and potential respiratory substrates. To test our hypothesis, we first screened *pgm* mutants and wild-type plants of four species for average differences in assimilate concentrations and δ^13^C values. We then investigated short-term ^13^C fractionation mechanisms in *N. sylvestris pgm* mutants and wild-type plants by analyzing the δ^13^C_OM_, δ^13^C_R_, and δ^13^C of individual compounds (e.g. sugars, starch, malate, and citrate) and their concentrations during a diel cycle. Finally, we measured the leaf gas exchange in both *N. sylvestris* genotypes to determine if potential δ^13^C differences caused by the PGM-knockout are influenced more by photosynthetic or post-photosynthetic ^13^C fractionations.

## Materials and methods

### Plant material

#### Experiment 1

**Fig. 2.**
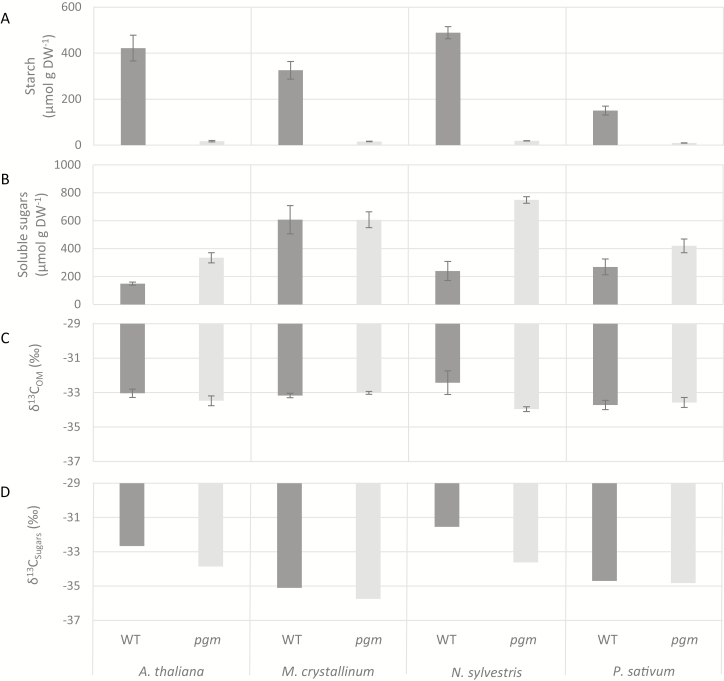
Screening of *pgm* mutants (experiment 1). Concentrations of starch (A) and soluble sugars (B), as well as δ^13^C values in organic matter (C) and sugars (D), measured in leaf discs at the end of the day in *pgm* mutants and wild-type plants of four species. Mean values ±1 SD are given (*n*=3).

We first screened wild-type and *pgm* genotypes of the four species *A. thaliana*, *M. crystallinum*, *N. sylvestris*, and *P. sativum*. All four species, including the facultative Crassulacean acid metabolism (CAM) plant *M. crystallinum*, use the C_3_ photosynthetic pathway (δ^13^C values less than –30‰, Fig. 2C). Plants were grown from seeds in a climate-controlled growth chamber. Light was provided during a 12 h photoperiod with a photosynthetic photon flux density (PPFD) of ~160 µmol m^–2^ s^–1^. Relative humidity was constantly at 70% during the day/night cycle, while temperature was 22 °C during the day and 18 °C during the night. Two weeks after germination, plantlets were transplanted into 160 ml pots filled with potting soil. Timing of sampling was adapted to the different growth habits of the four species but occurred during the exponential growth phase of each species, prior to flowering or pot limitation*. A.thaliana* and *M. crystallinum* plants were sampled 28 d after germination, while *P. sativum* and *N. sylvestris* were sampled after 17 d and 42 d, respectively. At the end of each sampling day, leaf discs (20 mm^2^) of three individuals were punched and transferred to reaction vials, frozen in liquid nitrogen to inactivate metabolism, and immediately stored at –80 °C.

#### Experiment 2

*N.sylvestris* wild-type and *pgm* mutant plants were grown from seeds under identical growth conditions to those in experiment 1. Two weeks after germination, plantlets were transplanted into 1 liter pots filled with potting soil. After 8 weeks, samples were taken over a 24 h period: fully expanded leaves from individual plants (*n*=3) were sampled after 0, 4, 8, 12, 14, 20, and 24 h, transferred to paper bags, and frozen in liquid nitrogen. In addition, aliquots of leaf dark-respired CO_2_ and climate chamber air were taken at each point in time (see methods below).

#### Experiment 3

For leaf gas exchange measurements, an additional batch of *N. sylvestris* wild-type and *pgm* mutant plants were grown under conditions identical to those described above. Measurements were performed at three points in time on individual *pgm* mutants and wild-type plants (*n*=5). Leaf material was sampled on the next day after 4 h of light, as described above.

### Isotope ratio analyses of leaf dark-respired CO_2_

Leaf dark-respired CO_2_ was sampled at the above-listed time points using the in-tube incubation method originally described by Werner *et al.* (2007) and modified by Lehmann *et al.* (2015). In short, leaf material was placed in 12 ml gas-tight glass vials (‘Exetainer’, Labco Ltd, Lampeter, Ceredigion, UK) and flushed with CO_2_-free synthetic air (Pangas, Dagmersellen, Switzerland). The exetainer was immediately darkened for 4 min, and subsequently an aliquot of the sample air (now with respired CO_2_) was transferred with a gas-tight syringe into a second exetainer pre-filled with dry N_2_ (N_2_ 5.0, Pangas). In addition, climate chamber air samples were taken and transferred to N_2_-filled exetainers. The gaseous samples in the exetainers were then analyzed using a modified Gasbench II (Zeeman *et al.*, 2008) connected to a Delta^Plus^XP isotope ratio mass spectrometer (Thermo-Fisher, Bremen, Germany) with a precision of ~0.1‰ (SD) for a quality control standard (400 ppm CO_2_ in artificial air).

### Isotope ratio analysis of organic compounds

#### Extraction and purification of organic compounds

Leaf samples from all experiments were freeze-dried (Beta 2-8 LD plus, Martin Christ, Osterode am Harz, Germany) and subsequently milled to a fine powder with a steel ball-mill (MM 200, Retsch, Haan, Germany) for further chemical analyses. Owing to biomass limitations, some of the leaf material from experiment 1 was pooled for δ^13^C analysis of sugars (Table 1).

**Table 1.** Comparison of modeled and observed δ^13^C values in *Nicotiana sylvestris pgm* mutants and wild-type (WT) plants

*Nicotiana sylvestris*	Experiment 3			Experiment 2					
genotype	*c*_i_	δ^13^C_M_	δ^13^C_OM_	δ^13^C_OM_	Sugars	Starch	Malate	Citrate	δ^13^C_R_
WT	164.9±29.4	–27.4±1.7	–27.6±0.2	–32.6±0.4	–32.2±0.7	–31.8±0.6	–24.4±2.1	–25.3±2.0	–28.9±2.5
*pgm*	204.3±28.9	–29.7±1.7	–28.7±0.6	–33.4±0.4	–34.3±0.7	–33.1±1.1	–26.3±1.6	–27.9±2.7	–30.3±2.3
*pgm* − WT	39.4	–2.3	–1.1	–0.8	–2.1	–1.3	–1.9	–2.6	–1.4

Modeled δ^13^C values of assimilates (δ^13^C_M_, ‰, Equation 1) derived from diel average *c*_i_ values (µmol mol^–1^) and observed δ^13^C values of organic matter (δ^13^C_OM_) are shown (all from experiment 3). The diel averages for δ^13^C_OM_, δ^13^C of dark-respired CO_2_ (δ^13^C_R_), and δ^13^C of different substrates are also given (all from experiment 2). For all parameters, the genotype difference (*pgm*−WT) is indicated. Mean values ±1 SD are shown.

For isotope analysis, 100 mg of dry leaf material was transferred into a 2 ml reaction vial and the water-soluble compounds (WSCs) were extracted in 1.5 ml of deionized water at 85 °C for 30 min in a water bath according to Lehmann *et al.* (2015). After centrifugation (10 000 *g*, 2 min), the supernatant holding the WSC fraction was transferred into a new reaction vial and stored at –20 °C for further purification, while the pellet was used for isolation of starch (see below). Soluble carbohydrates (‘sugars’) and the bulk organic acid fractions were isolated from the WSC fraction by ion-exchange chromatography, as described by Lehmann *et al.* (2015). In brief, Dowex 50WX8 in hydrogen form and Dowex 1X8 in formate form (both 100–200 mesh, Sigma-Aldrich, Buchs, Switzerland) were packed in 5 ml syringes (B. Braun, Melsungen, Germany) arranged in a custom-built rack so that the outlet of the cation exchanger (Dowex 50WX8) syringe was connected to the inlet of the anion exchanger (Dowex 1X8) syringe. After extensive flushing of the Dowex material with deionized water, the WSC fraction (~1 ml) was added to the upper ion exchanger and the neutral sugar fraction was eluted with 30 ml of deionized water. Subsequently, the organic acid fraction was eluted from the anion exchanger with 35 ml of 1 M HCl. All aqueous and acidic samples were frozen at –20 °C and freeze-dried (Hetosicc CD 52-1, Heto, Birkerød, Denmark). The remaining pellet was dissolved in 1 ml of deionized water. All samples were stored at –20 °C until isotope analysis.

Leaf starch was isolated from the remainder of the hot water extraction by enzymatic hydrolysis (Richter *et al.*, 2009; Lehmann *et al.*, 2015). In short, the pellet was washed several times with 1.5 ml of a methanol/chloroform/water (MCW) solution and subsequently with deionized water, and then bench-dried overnight. On the next day, the pellet was additionally oven-dried at 60 °C for 1 h to fully remove chloroform residues, suspended in deionized water, and boiled at 100 °C for 15 min in a water bath to gelatinize starch. The starch was hydrolyzed with a heat-stable α-amylase (EC 3.2.1.1, Sigma-Aldrich) at 85 °C for 2 h and then centrifuged (10 000 *g*, 2 min). The supernatant was freed from enzymatic residues by using centrifugation filters (Vivaspin 500, Sartorius, Göttingen, Germany) and stored in 2 ml reaction vials at –20 °C until isotope analysis.

#### δ^13^C values of bulk leaf organic matter, sugars, and starch

δ^13^C analysis of bulk organic matter, sugars, and starch was performed using an elemental analyzer (Flash, ThermoFisher) coupled to a Delta^Plus^XP isotope ratio mass spectrometer (Werner *et al.*, 1999; Brooks *et al.*, 2003). Leaf material was weighed into Sn capsules (5×9 mm, Säntis, Teufen, Switzerland), and aliquots of solubilized sugars and starch were pipetted into the Sn capsules and oven-dried at 60 °C. Positioning of the samples, blanks, and laboratory standards, as well as the referencing of the measurement, was done as suggested by Werner and Brand (2001). Measurement precision of a long-term quality control standard was typically better than 0.2‰ (SD). The applied chemical isolation methods were shown to be free of isotope fractionation by analysis of commercial standard materials with respect to the measurement precision.

#### δ^13^C values and concentrations of individual organic acids

The analysis of δ^13^C values and concentrations of individual organic acids (citrate and malate) was performed by coupling an Isolink HPLC device to a DeltaV isotope ratio mass spectrometer (all Thermo-Fisher; Lehmann *et al.*, 2015, 2016*b*). Before analysis, the aqueous bulk organic acid fractions have to be passed through a 0.45 µm syringe filter (Infochroma, Zug, Switzerland). Organic acids were separated on a 4.6×300 mm Allure Organic Acids column (Restek, Bellefonte, PA, USA) at 8 °C using 100 mM KH_2_PO_4_ (pH 3) as a mobile phase with a flow rate of 500 µl min^–1^. All organic compounds were oxidized to CO_2_ at 99 °C using Na_2_S_2_O_8_ under acidic conditions. The CO_2_ was subsequently separated from the mobile phase and measured for its isotope ratio and concentration in the IRMS. Every 10 samples, a set of malate and citrate laboratory standards of different concentrations (10–180 ng C μl^–1^) was analyzed. Offset corrections to EA-IRMS δ^13^C values and determination of concentrations were performed according to Rinne *et al.* (2012). The applied method was shown to be free of isotope fractionation by analysis of standard materials with respect to the measurement precision, which was typically better than 0.4‰ (SD).

### Sugar and starch concentration measurements

Soluble sugars were directly extracted from leaf discs (experiment 1) or 10 mg of leaf powder (experiment 2) by two sequential additions of 80% ethanol and one addition of 50% ethanol, where the supernatant was recovered and pooled. During each extraction, the sample was heated to 80 °C for 30 min. Sugars were photometrically measured according to Mueller-Roeber *et al*. (1992). Starch in the remaining pellet was extracted and photometrically measured as described in Hostettler *et al.* (2011). Sugar and starch concentrations are presented in μmol glucose equivalents per mg dry mass calculated using a pre-determined conversion factor.

### Leaf gas exchange measurements

A cuvette with a measurement area of 6 cm^2^ coupled to a Licor 6400 was used for leaf gas exchange measurements (all supplied by LI-COR, Lincoln, NE, USA). Temperature and relative humidity conditions in the cuvette were 21.9±0.5 °C and 63.9±7.3%, respectively, throughout all diurnal measurements. A flow rate of 250 µmol s^–1^, CO_2_ concentration of 400 µmol mol^–1^ CO_2_, PPFD of 160 µmol m^–2^ s^–1^, and leaf temperature of 22.0 °C were constantly maintained. Climatic and light conditions in the cuvette were thus nearly identical to growth conditions.

### Data analysis and calculations

Leaf gas exchange data were used to model δ^13^C values of recent assimilates (δ^13^C_M_). The calculation was based on standard ^13^C discrimination models (Farquhar *et al.*, 1982):

$$\begin{array}{l} \delta^{13}C_{M}=\delta^{13}C_{Air}-\left(a+\left(b-a\right)*c_{i}/c_{a}\right) \end{array}$$ (1)

where δ^13^C_Air_ reflects the diel average of climate chamber air (–13.7±1.2‰, mean ±1 SD), *a* stands for the diffusional isotope fractionation (4.4‰), *b* for the enzymatic isotope fractionation (27‰, mainly due to Rubisco-catalyzed carboxylation reactions), and *c*_i_/*c*_a_ is the ratio of leaf internal and atmospheric CO_2_ concentrations (*c*_a_ was held constant at 400 µmol mol^-1^).

The diel apparent respiratory ^13^C fractionation was calculated as:

$$\begin{array}{l} e=\delta^{13}C_{Substrate}-\delta^{13}C_{R} \end{array}$$ (2)

where δ^13^C_Substrate_ is the average δ^13^C value of a respiratory substrate, and δ^13^C_R_ is the average δ^13^C value of leaf dark-respired CO_2_ during a 24 h diel cycle.

Linear mixed effects models were used to test the individual effects of species and genotype (experiment 1) or genotype and time (experiment 2) on gas exchange, δ^13^C values, and concentrations, as well as interactions between these effects. If no significant interaction between individual effects was found, the model was repeated without the interaction term. The individual plant ID was included as a random effect. Effects with *P*-values <0.05 were considered to be statistically significant. Where not indicated otherwise, all given errors denote standard errors. All statistical analyses were performed in R version 3.4.4. (R Core Team, 2018).

## Results

### Experiment 1: screening of *pgm* mutants and wild-type plants

Functioning of the PGM-knockout in all screened species was demonstrated by clearly lower starch concentrations in *pgm* mutants than in wild-type plants (Fig. 2A). Sugar concentrations were generally higher in *pgm* mutants than in wild-type plants, with the greatest difference in *N. sylvestris* plants, while only *M. crystallinum* plants showed little difference in sugar concentrations (Fig. 2B). Isotope analysis of leaf bulk organic matter showed a clear δ^13^C difference of 1.6‰ between *pgm* and the wild type for *N. sylvestris* plants (Fig. 2C) but not for other species. A similar tendency was found for δ^13^C values of sugars (Fig. 2D), with the largest differences being up to 2.0‰ for sugars of *A. thaliana* and *M. crystallinum*. Whether the results were statistically significant for each species could not be determined because all three replicates were pooled for the isotope analysis of sugars. In summary, the effect of the PGM-knockout was greatest for δ^13^C_OM_ and δ^13^C values of sugars in *N. sylvestris* plants, which also showed the largest difference in sugar and starch concentrations between the two genotypes.

### Experiment 2: diel cycle of *N. sylvestris pgm* mutants versus wild-type plants

**Fig. 3.**
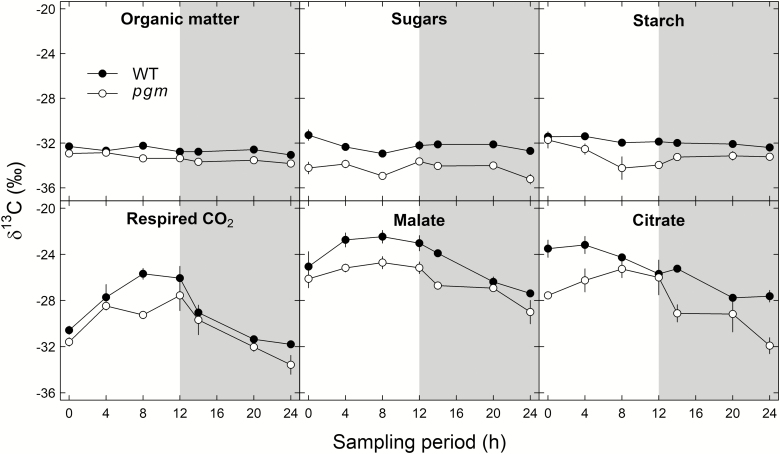
Diel δ^13^C variation (‰) in dark-respired CO_2_ and substrates in leaves of *Nicotiana sylvestris* wild-type plants (WT; filled circles) and *pgm* mutants (experiment 2; open circles). The gray shaded area indicates night-time. Error bars can be smaller than data point symbols. Mean values ±1 SE are given (*n*=2–3).

Given the above-mentioned results, we further investigated the mechanisms of ^13^C fractionation in *N. sylvestris pgm* mutants and wild-type plants by measuring δ^13^C values of leaf bulk organic matter (δ^13^C_OM_), assimilates, organic acids, and dark-respired CO_2_ (δ^13^C_R_) during a diel cycle (Fig. 3; Table 1). In general, we observed clear effects of time and genotype for δ^13^C_OM_ and δ^13^C_R_, and for δ^13^C values of all substrates (*P*<0.001 for both time and genotype). δ^13^C_OM_ values, ranging from –32.2 to –33.8 for both genotypes and all points in time, were on a diel average 0.8‰ more negative in *pgm* mutants than in wild-type plants. δ^13^C values of sugars and starch, ranging from –31.3‰ to –35.2‰ for both genotypes and all points in time, were on a diel average 2.1‰ and 1.3‰ more negative in *pgm* mutants than in wild-type plants. On a diel average, starch was 0.4‰ and 1.2‰ ^13^C enriched compared with sugars for wild-type plants and *pgm* mutants, respectively.

In comparison with the δ^13^C values of plant assimilates, the δ^13^C_R_ and δ^13^C values of malate and citrate were less negative (except for some δ^13^C values at 0, 20, and 24 h) and showed wider variation during the diel cycle for both genotypes (Fig. 3; Table 1). δ^13^C_R_ values ranging from –25.7‰ to –33.6‰ increased by 4.3‰ during the day and decreased by 5.9‰ during the night for both genotypes. δ^13^C_R_ values were on a diel average ~1.4‰ more negative in *pgm* mutants than in wild-type plants, with the exception that after 8 h of illumination δ^13^C_R_ values were 3.6‰ more negative. δ^13^C values of malate and citrate, ranging from –22.5‰ to –31.9‰ for both genotypes and all points in time, were on a diel average 1.9‰ and 2.6‰ more negative for *pgm* mutants than for wild-type plants. δ^13^C values of malate showed an increase of 1‰ for *pgm* mutants and 2‰ for wild-type plants during the day, while a similar decrease of 4.1‰ during the night was observed for both genotypes. Also, δ^13^C values of citrate for *pgm* mutants showed an increase of 1.9‰ during the day and a decrease of 5.9‰ during the night, while the values for wild-type plants steadily decreased by 4.1‰ during the diel cycle. Thus, δ^13^C_R_ and δ^13^C values of all substrates were clearly more negative for *pgm* mutants than for wild-type plants.

**Fig. 4.**
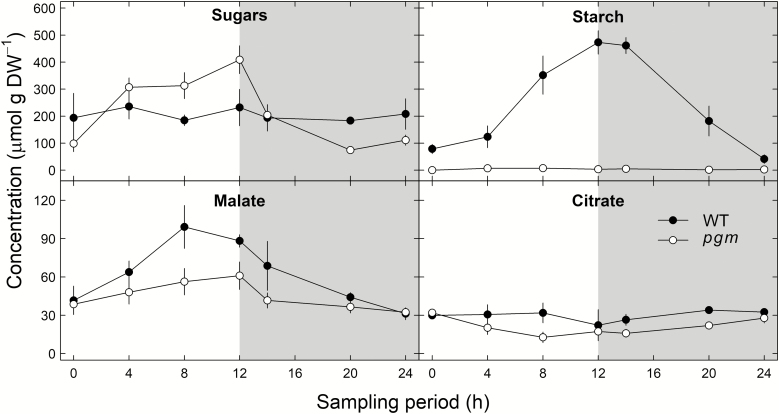
Diel variation in substrate concentrations (µmol g DW^–1^) in leaves of *Nicotiana sylvestris* wild-type plants (WT; filled circles) and *pgm* mutants (experiment 2; open circles). Sugar and starch concentrations are normalized to hexose units to facilitate comparison. The gray shaded area indicates night-time. Please note the *y*-axis scale differences between the upper and lower panels. Error bars can be smaller than data point symbols. Mean values ±1 SE are given (*n*=2–3).

We also measured assimilate and organic acid concentrations during a diel cycle in both *pgm* mutants and wild-type *N. sylvestris* plants (Fig. 4). As expected, the *pgm* mutant showed only very low diel starch concentrations, while wild-type plants showed a clear diel cycle, with the lowest and highest concentrations occurring at the beginning and end of the day (*P*<0.001 for the interaction between time and genotype). In contrast, sugar concentrations showed a clear diel cycle in *pgm* mutants, with values up to 76% higher during the day and up to 53% lower during the night compared with values in wild-type plants, which showed no clear diel cycle (*P*<0.05 for the interaction between time and genotype). Moreover, malate and citrate concentrations were both on average 28.5% lower in *pgm* mutants than in wild-type plants during the diel cycle (*P*<0.01). Malate concentrations were highest at the end of the day (nearly twice higher at 8 h in wild-type plants compared with concentrations in *pgm* mutants) and lowest at the end of the night in both genotypes (*P*<0.001), while citrate concentrations showed no distinct diel cycle in either genotype (*P*>0.05). Thus, both assimilate and organic acid metabolism were strongly influenced by the *PGM*-knockout.

### Experiment 3: leaf gas exchange measurements and modeling of δ^13^C_M_

**Fig. 5.**
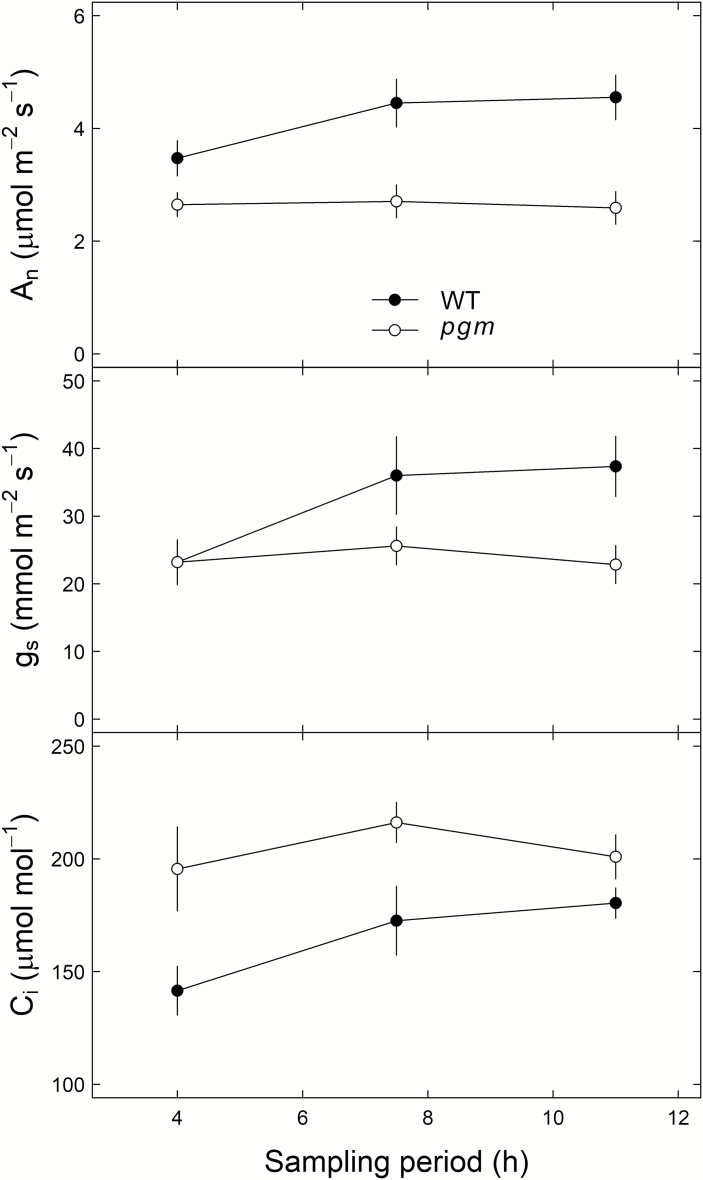
Leaf gas exchange measurements in *Nicotiana sylvestris* wild-type plants (WT; filled circles) and *pgm* mutants (open circles) during a 12 h light period (experiment 3). *A*_n_, net assimilation rate (µmol m^–2^ s^–1^), *g*_s_, stomatal conductance (mmol m^–2^ s^–1^), and *C*_i_, intercellular CO_2_ concentration (µmol mol^–1^). Mean values ±1 SE are given (*n*=5).

The PGM-knockout affected the assimilation rate (*A*_n_; *P*<0.001) and stomatal conductance to water vapor (*g*_s_; *P*<0.05), causing up to 2.0 μmol m^–2^ s^–1^ and 14.5 mmol m^–2^ s^–1^ lower values, respectively, in *pgm* mutants than in wild-type plants of *N. sylvestris* (Fig. 5). Although the genotype differences in *A*_n_ and *g*_s_ tended to increase during the diurnal cycle, no clear significant temporal variations during the light period were observed (*P*>0.05). The changes in *A*_n_ and *g*_s_ caused up to 54.0 µmol mol^–1^ higher intercellular CO_2_ concentrations (*c*_i_) in *pgm* mutants than in wild-type plants (*P*<0.001), but again without clear temporal variation (*P*>0.05).

The *c*_i_ values derived from leaf gas exchange measurements were used to model the δ^13^C of assimilates (δ^13^C_M_; Table 1, Equation 1). δ^13^C_M_ values of both genotypes were on a diel average consistent with the measured δ^13^C_OM_ values of wild-type plants from the same experiment. δ^13^C_OM_ differences between experiment 2 and 3 were probably caused by temporal δ^13^C differences in the climate chamber air. In particular, fossil fuel CO_2_ emissions in winter have most probably caused lower δ^13^C_Air_ and thus lower δ^13^C_OM_ for plants of experiment 2 than for those of experiment 3, which was conducted in summer. The genotype difference in δ^13^C_M_ of 2.3‰ was similar to that for sugars (2.1‰), malate (1.9‰), and citrate (2.6‰), but tended to be greater than the difference observed for δ^13^C_OM_ (0.8–1.1‰) and δ^13^C_R_ (1.4‰). Thus, the genotype difference in both modeled and observed δ^13^C values showed a consistent ^13^C depletion of *pgm* mutants compared with wild-type plants across all experiments.

### Apparent respiratory ^13^C fractionation and correlations with δ^13^C of substrates

**Fig. 6.**
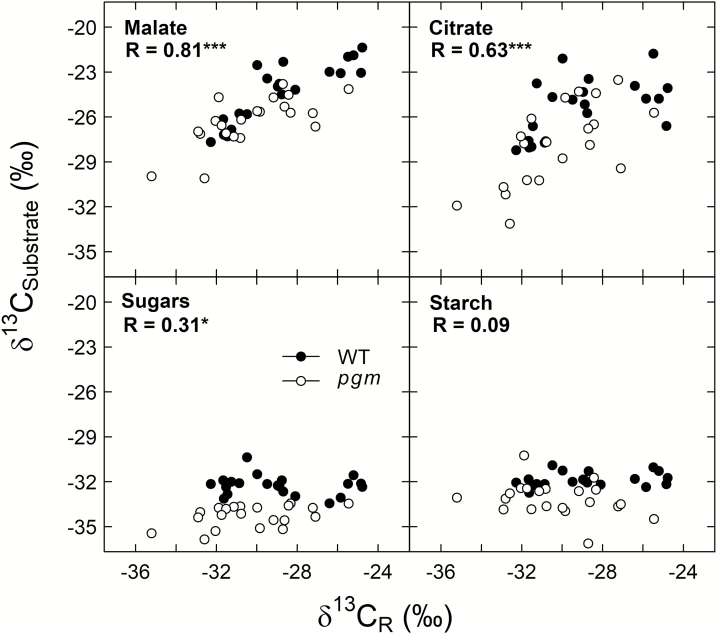
Correlations between δ^13^C values of dark-respired CO_2_ (δ^13^C_R_) and different respiratory substrates (δ^13^C_Substrate_) in leaves of *Nicotiana sylvestris* wild-type plants (WT; filled circles) and *pgm* mutants (open circles) for both genotypes and all points in time. Pearson correlation coefficients (*R*) and *P*-values (***P <0.001, *P <0.05) are given.

**Fig. 7.**
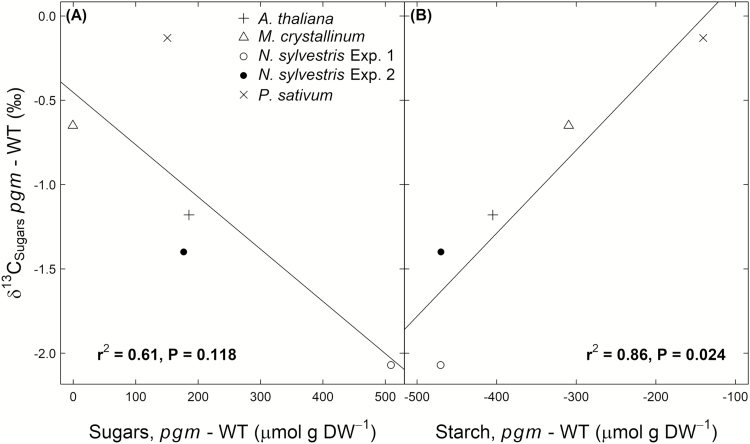
Relationships between genotype differences (*pgm*−WT) in δ^13^C values of sugars (δ^13^C_Substrate_, ‰) and sugar (A) and starch (B) concentrations in leaves of *Nicotiana sylvestris* at the end of the day. The coefficient of determination (*r*^2^) and *P*-value are given in each case, based on linear regression (solid line).

We calculated the diel average apparent respiratory ^13^C fractionation using Equation 2. The *e* values differed among the potential respiratory substrates for both genotypes (Table 2), with the highest values observed for malate and citrate (up to 4.5‰) and the lowest values observed for sugars, starch, and bulk organic matter (up to –3.9‰). No clear significant differences were observed between the genotypes for any substrate (*P*>0.05, *t*-test). In addition, we investigated the correlation between individual δ^13^C_R_ values and the δ^13^C values of different substrates for the two genotypes and all points in time (Fig. 6). We found a strong correlation for malate (*R*=0.81, *P*<0.001) and citrate (*R*=0.63, *P*<0.001) but a weaker correlation for sugars (*R*=0.31, *P*<0.05) and no correlation for starch (*R*=0.09, *P*>0.05). An analysis of covariance (ANCOVA) showed that the relationship between δ^13^C_R_ and each δ^13^C_Substrate_ was not influenced by the PGM-knockout (*P*>0.05). In addition, genotype differences across the four species in the δ^13^C of sugars were found to be negatively related to a genotype difference in sugar concentrations (*r*^2^=0.61, *P*=0.118) and positively related to those in starch concentrations (*r*^2^=0.86, *P*=0.024; Fig. 7).

**Table 2.** Average diel apparent respiratory ^13^C fractionation (*e*, Equation 2) for different potential respiratory substrates in leaves of *Nicotiana sylvestris* wild-type and *pgm* mutant plants (all from experiment 2)

Substrate	*e* (‰)	
	Wild type	*pgm*
Organic matter	–3.7±0.5	–3.1±0.5
Sugars	–3.4±0.6	–3.9±0.5
Starch	–3.1±0.5	–2.8±0.6
Malate	4.5±0.3	4.0±0.4
Citrate	3.6±0.3	2.4±0.4

No significant differences between genotypes for any substrate were observed (*t*-test, *P*>0.05). Mean values ±1 SE are given (*n*=19–21).

## Discussion

Our screening (experiment 1) confirmed that *pgm* mutants had lower starch concentrations but higher sugar concentrations at the end of the day compared with wild-type plants for most species (Fig. 2A, B), clearly demonstrating that the primary assimilate metabolism was affected by the PGM-knockout across all species. As shown for *N. sylvestris* plants (experiment 2), the sugar pool in *pgm* mutants was used up during the night, similar to the starch pool in wild-type plants for various biosynthetic and respiratory processes (Fig. 4). This demonstrates that the PGM-knockout leads to a re-routing of freshly assimilated triose phosphates towards cytosolic sugar biosynthesis, as plastidic starch biosynthesis is prevented in mutant plants (Streb and Zeeman, 2012). Thus, the lack of starch as an important temporary carbon sink and chemical energy buffer in *pgm* mutants is at least partially counterbalanced by higher sugar biosynthesis. The *pgm* mutant therefore has great potential as a tool to study ^13^C fractionation responses related to changes in the diel starch metabolism.

### On the post-photosynthetic ^13^C fractionation via pFBA in response to *pgm*-induced starch deficiency

We hypothesized that post-photosynthetic ^13^C fractionation via pFBA plays an important role in the δ^13^C variation in plant material. We expected that, owing to the blocked starch biosynthesis in *pgm* mutants, the equilibrium isotope effect on the pFBA reaction would be only slightly or not at all expressed (Fig. 1). Given that more triose phosphates are exported to the cytosol under such conditions, more ^13^C enriched sugars and, by extension, more ^13^C enriched organic matter should be produced in *pgm* mutants than in wild-type plants. However, carbon isotope analysis of leaf organic matter and sugars of the four species revealed, in contrast to our hypothesis, either no clear δ^13^C differences or a ^13^C depletion in *pgm* mutants compared with wild-type plants of *N. sylvestris* (Fig. 2C, D). This shows that the pFBA-related post-photosynthetic ^13^C fractionation is probably not the main reason for the observed δ^13^C differences between the genotypes.

Theoretically, only one out of six triose phosphate molecules after CO_2_ fixation is used for sugar/starch synthesis, while the other five molecules enter the regeneration part of the Calvin–Benson–Bassham cycle. The PGM-knockout might therefore affect the kinetic operation conditions of the pFBA reaction much less than expected. For example, the ^13^C enrichment of starch compared with sugars was 0.8‰ greater on a diel average in *pgm* mutants than in wild-type plants (Fig. 3). This is a surprising result given that the main route of starch biosynthesis is blocked by the PGM-knockout in the mutant plant. The ^13^C enrichment of the starch residue compared with that of sugars in *pgm* mutants must therefore be caused by a different biosynthetic pathway such as a cytosolic bypass reaction. Higher glucose-6-phosphate concentrations in chloroplasts of *pgm* mutants than in those of wild-type plants have been observed (Kofler *et al.*, 2000). Assuming that pFBA is operating normally in *pgm* mutants, glucose-6-phosphate molecules could still show the typical ^13^C enrichment induced by pFBA. Transport of glucose-6-phosphate to the cytosol via the glucose-6-phosphate translocator in connection with the glucose-1-phosphate transport from the cytosol to the chloroplast (Fettke *et al.*, 2011) would mean that the plastidic PGM could be bypassed by the cytosolic isoenzyme. Such a mechanism might explain why starch is more ^13^C enriched compared with sugars in the *pgm* mutant compared with wild-type plants.

The low temporal variation in the δ^13^C of starch and sugars is in line with observations that there is no clear circadian rhythm in δ^13^C of leaf bulk sugars and starch of other plant species (Sun *et al.*, 2009; Lehmann *et al.*, 2016*b*). However, this result contradicts previous findings that starch-related post-photosynthetic ^13^C fractionations influence (e.g. via pFBA) short-term δ^13^C variation in leaf and phloem assimilates (Gessler *et al.*, 2008; Kodama *et al.*, 2011; Lehmann *et al.*, 2015). These opposing observations stress that species-specific differences need to be considered when modeling temporal δ^13^C variation in assimilates, including the extent of isotope effects on enzymatic reactions, compartmentalization of sugar/starch pools, and their pool sizes and turnover rates. In summary, potential post-photosynthetic ^13^C fractionation via pFBA cannot explain the observed δ^13^C difference between *pgm* mutants and wild-type plants.

### *pgm*-induced starch deficiency affects daytime organic acid metabolism but not apparent respiratory ^13^C fractionations

The organic acid metabolism (i.e. δ^13^C values and concentrations of malate and citrate) and δ^13^C_R_ were clearly affected by the PGM-knockout*-*induced starch deficiency (Figs 3, 4). Both organic acids were generally ^13^C enriched compared with starch and sugars of both genotypes. This pattern can be explained by the activity of the phosphoenolpyruvate carboxylase (PEPC) reaction that catalyzes the conversion of phosphoenolpyruvate and hydrogen carbonate to oxaloacetate with a net isotope discrimination against ^13^C of –5.7‰ relative to CO_2_ in equilibrium with hydrogen carbonate (Farquhar *et al.*, 1989). The ^13^C enriched oxaloacetate functions as a precursor for malate and citrate. The PEPC reaction is therefore assumed to reflect an anaplerotic flux in C_3_ plants that replenishes withdrawn carbon skeletons from the tricarboxylic acid (TCA) cycle (Werner *et al.*, 2011; Lehmann *et al.*, 2015). However, it is generally accepted that the TCA cycle is not fully functional during the day due to light inhibition of key enzymes (Hanning and Heldt, 1993; Tcherkez *et al.*, 2005; Sweetlove *et al.*, 2010). Given the light-induced limitation of the TCA cycle, the light-activated PEPC reaction (together with the non-inhibited malate dehydrogenase reaction) must be responsible for the often-observed accumulation of malate (via oxaloacetate) during the day (Scheible *et al.*, 2000; Gessler *et al.*, 2009; Igamberdiev and Bykova, 2018). In fact, we observed a simultaneous increase in δ^13^C values and concentration of malate for both *N. sylvestris* genotypes, although these increases were lower in *pgm* mutants than in wild-type plants (Figs 3, 4). This strongly suggests that the anaplerotic PEPC flux is down-regulated in *pgm* mutants during the day, potentially owing to the increase in sugar concentration which supplies glycolysis and the TCA cycle with additional carbon skeletons (Figs 2, 4).

Moreover, we observed that diel δ^13^C_R_ values were strongly related to δ^13^C values of malate and citrate for both genotypes but were weakly or not at all correlated with δ^13^C values of sugars and starch, respectively (Fig. 6). This result is in line with findings from previous studies that malate is a key substrate for leaf dark-respired CO_2_ (Gessler *et al.*, 2009; Lehmann *et al.*, 2015, 2016*b*), particularly shortly after darkening, as shown in an experiment with position-specific ^13^C-labeled malate (Lehmann *et al.*, 2016*a*). However, despite the lower daytime δ^13^C values and concentrations of malate in *N. sylvestris pgm* compared with wild-type plants (Figs 3, 4), we generally observed no genotype differences in the average diel apparent respiratory ^13^C fractionation for various potential substrates (Table 2) or in the δ^13^C relationships between dark-respired CO_2_ and substrates (Fig. 6). Thus, starch deficiency induced by the PGM-knockout had no clear influence on apparent respiratory ^13^C fractionations.

### *pgm*-induced starch deficiency causes photosynthetic ^13^C fractionations

Interestingly, the differences in sugar and starch concentrations between genotypes were related to the δ^13^C difference in sugars across all species (Fig. 7), with the largest δ^13^C difference corresponding to the largest differences in sugar and starch concentrations between *N. sylvestris* genotypes. This suggests that the changes in the assimilate pool cause ^13^C fractionations. It has been demonstrated in several studies that an increase in sugar concentrations (as observed in *pgm* mutants) decreases the photosynthetic activity of a plant (Krapp and Stitt, 1995; Paul and Driscoll, 1997; Burkle *et al.*, 1998). This hypothesis is supported by our gas exchange measurements, which showed lower *A*_n_ and *g*_s_ values and higher *c*_i_ values in *pgm* mutants than in wild-type *N. sylvestris* plants during the day (Fig. 5). In particular, the lower assimilation rates in *pgm* mutants are widely supported by findings in *A. thaliana* and *N. sylvestris* plants (Caspar *et al.*, 1985; Huber and Hanson, 1992; Geiger *et al.*, 1995; Sun *et al.*, 1999). Thus, the changes in starch and sugar pool sizes in response to the PGM-knockout (Fig. 7) have influenced leaf gas exchange and thus caused differences in photosynthetic ^13^C fractionations, explaining the observed ^13^C depletion in the organic matter, sugars, organic acids, and dark-respired CO_2_ in *pgm* mutants (Figs 2, 3).

To determine whether photosynthetic ^13^C fractionations are actually the main driver of the δ^13^C differences observed between the *N. sylvestris* genotypes, we modeled δ^13^C values of assimilates from leaf gas exchange measurements and found that δ^13^C_M_ and δ^13^C_OM_ values from the same experiment were in good agreement (Table 1). Most importantly, the δ^13^C_M_ difference between the two genotypes of 2.3‰ was similar to the differences observed for sugars and organic acids. This again indicates that δ^13^C differences in fresh assimilates caused by the *pgm*-induced starch deficiency are primarily driven by photosynthetic ^13^C fractionations. Further, the smaller genotype difference for δ^13^C_OM_ of 0.8–1.1‰ compared with the difference for δ^13^C_M_ might be explained by structural components in organic matter. For example, cellulose reflects and integrates all leaf gas exchange variation that occurs during the period of growth or leaf expansion. Given that *pgm* mutants grow differently from wild-type plants (Huber and Hanson, 1992), the leaf gas exchange and thus the photosynthetic ^13^C fractionations that have shaped the δ^13^C of cellulose and organic matter might differ from those that have shaped the δ^13^C of assimilates measured at the end of the growth period. In addition, potential isotope fractionations related to sugar and starch biosynthesis may cancel each other out during the diel buildup of organic matter, reducing the genotype differences in δ^13^C_OM_ compared with δ^13^C_M_. Overall, we conclude that the majority of δ^13^C genotype differences can be primarily explained by leaf gas exchange adaptations and thus by *c*_i_/*c*_a_-driven photosynthetic ^13^C fractionations, while potential post-photosynthetic ^13^C fractionations in response to the PGM-knockout are of minor importance.

### Conclusions and implications

Through this investigation, we showed that the δ^13^C values of organic matter, substrates, and dark-respired CO_2_ are strongly influenced by differences in leaf gas exchange and corresponding photosynthetic ^13^C fractionations. Changes in assimilate pool sizes due to the PGM-knockout may have induced the photosynthetic ^13^C fractionations by suppressing the photosynthetic activity of the mutant plants. δ^13^C variability in plant material might therefore be indicative of increases in sugar concentrations or changes in the sugar/starch ratio. It is likely that the observed shift in δ^13^C due to starch deficiency might also occur in non-genetically modified plants in response to changes in environmental conditions (e.g. drought) or nutrient availability; this topic should be investigated in future studies.

Moreover, we demonstrated that post-photosynthetic ^13^C fractionation (e.g. via pFBA or respiration) is not or only slightly affected by *pgm*-induced starch deficiency. Instead the isotopic signature of both plant mutants and wild-type plants is dominated by the photosynthetic ^13^C fractionation processes. This might have important implications for the reconstruction of leaf gas exchange responses to climatic conditions of the past using δ^13^C values of plant compounds or other biomarkers (Ehleringer *et al.*, 1997; Gessler *et al*., 2014).

Finally, we conclude that δ^13^C changes in plant material may not only contain information on physiological and biochemical processes but might also be helpful for inferring and reconstructing genetic responses. Our findings might therefore be interesting for retrospective studies on tree decline and mortality using tree-ring growth patterns in combination with stable isotope analysis of carbon, oxygen, and hydrogen (Scheidegger *et al.*, 2000; Cormier *et al.*, 2018; Gessler *et al.*, 2018) and with genome analysis (Heer *et al.*, 2018). Our mutant approach thus paves the way for future studies exploring the biochemical and genetic background of isotope fractionations in plants.
